# Alcohol Metabolism Potentiates HIV-Induced Hepatotoxicity: Contribution to End-Stage Liver Disease

**DOI:** 10.3390/biom9120851

**Published:** 2019-12-10

**Authors:** Murali Ganesan, Moses New-Aaron, Raghubendra Singh Dagur, Edward Makarov, Weimin Wang, Kusum K. Kharbanda, Srivatsan Kidambi, Larisa Y. Poluektova, Natalia A. Osna

**Affiliations:** 1 Research Service, Veterans Affairs Nebraska-Western Iowa Health Care System, Omaha, NE 68105, USA; murali.ganesan@unmc.edu (M.G.); moses.newaaron@unmc.edu (M.N.-A.); Raghu.Dagur@unmc.edu (R.S.D.); KKharbanda@unmc.edu (K.K.K.); 2 Department of Internal Medicine, University of Nebraska Medical Center, Omaha, NE 68105, USA; 3 Department of Environmental, Agriculture and Occupational Health, College of Public Health, University of Nebraska Medical Center, Omaha, NE 68105, USA; 4 Department of Pharmacology and Experimental Neuroscience, University of Nebraska Medical Center, Omaha, NE 68105, USA; Makarove@unmc.edu (E.M.); Weimin.Wang@unmc.edu (W.W.); lpoluekt@unmc.edu (L.Y.P.); 5 Department of Chemical and Biomolecular Engineering, University of Nebraska, Lincoln, NE 68588, USA; skidambi2@unl.edu

**Keywords:** human immunodeficiency virus, hepatocytes, ethanol, acetaldehyde, apoptotic bodies, inflammation, fibrosis

## Abstract

In an era of improved survival due to modern antiretroviral therapy, liver disease has become a major cause of morbidity and mortality, resulting in death in 15–17% of human immunodeficiency virus (HIV)-infected patients. Alcohol enhances HIV-mediated liver damage and promotes the progression to advanced fibrosis and cirrhosis. However, the mechanisms behind these events are uncertain. Here, we hypothesize that ethanol metabolism potentiates accumulation of HIV in hepatocytes, causing oxidative stress and intensive apoptotic cell death. Engulfment of HIV-containing apoptotic hepatocytes by non-parenchymal cells (NPCs) triggers their activation and liver injury progression. This study was performed on primary human hepatocytes and Huh7.5-CYP cells infected with HIV-1ADA, and major findings were confirmed by pilot data obtained on ethanol-fed HIV-injected chimeric mice with humanized livers. We demonstrated that ethanol exposure potentiates HIV accumulation in hepatocytes by suppressing HIV degradation by lysosomes and proteasomes. This leads to increased oxidative stress and hepatocyte apoptosis. Exposure of HIV-infected apoptotic hepatocytes to NPCs activates the inflammasome in macrophages and pro-fibrotic genes in hepatic stellate cells. We conclude that while HIV and ethanol metabolism-triggered apoptosis clears up HIV-infected hepatocytes, continued generation of HIV-expressing apoptotic bodies may be detrimental for progression of liver inflammation and fibrosis due to constant activation of NPCs.

## 1. Introduction

Several hepatobiliary disorders, as well as overt liver injury, are associated with human immunodeficiency virus (HIV) infection. In an era of improved survival due to modern antiretroviral therapy (ART), liver disease has become a major cause of morbidity and mortality among HIV-infected persons, resulting in death in 15–17% of HIV-infected patients [1]. Modern ART efficiently blocks active HIV replication in HIV-permissive cells but does not eliminate latently infected cells that have HIV-DNA integrated into the host genome, thereby improving disease outcomes without halting liver disease progression [2]. The recognition of HIV-induced liver injury as an important clinical manifestation was supported by many clinical studies [3,4,5], with special emphasis on association between HIVRNA content and liver fibrosis development [6,7]. While HIV monoinfection by itself elevates liver transaminase levels, the exacerbation of liver injury is even more prominent when HIV patients are either co-infected with hepatotropic viruses, hepatitis C virus (HCV), and hepatitis B virus (HBV), or are exposed to liver-damaging substances such as alcohol [8]. These insults serve as the potent second hits in acceleration of HIV-induced liver disease [1,9,10].

Alcohol is a potent trigger of HIV-mediated liver damage, which frequently ends in progression to advanced fibrosis and cirrhosis [10,11]. The mechanisms behind these events are uncertain. They may be partially attributed to changes in gut microbiota and enhanced gut leakage induced by both HIV and alcohol, causing liver non-parenchymal cells activation via the Toll-like receptor (TLR) 4 pathway [12,13]. However, HIV-alcohol-mediated liver disease is not limited only to one mechanism. Furthermore, while HIV interaction with multiple hepatic cell types, including Kupffer cells (KCs) and hepatic stellate cells (HSCs), has been already reported [14,15,16], the detailed evaluation of HIV infection in hepatocytes as well as mechanisms by which ethanol metabolism triggers liver inflammation and fibrosis require further clarification. 

Although liver injury in HIV patients was previously attributed to ART-related hepatotoxicity [17] and that now new medications without significant hepatotoxic properties have become available [18], it is clear that HIV by itself may induce hepatocyte death. The ability of HIV to trigger apoptosis is well-documented in CD4+ lymphocytes [19]. However, other studies indicate that apoptosis predominantly occurs in bystander, not productively infected, lymph node cells [20]. To our knowledge, the effects of ethanol metabolism on HIV-induced apoptosis of hepatocytes as well as its relation to infection of these cells with HIV had not been investigated. Hepatocytes are major sites of ethanol metabolism and comprise 80% of liver cells. Thus, the pro-apoptotic effects of HIV potentiated by ethanol metabolism are crucial for liver injury even in the absence of ART. Here, we hypothesize that ethanol metabolism potentiates accumulation of HIV in hepatocytes, causing oxidative stress and intensive apoptotic cell death. Engulfment of HIV-containing apoptotic hepatocytes by non-parenchymal cells promotes their activation and liver injury progression. Our findings indicate that ethanol metabolism and mainly, acetaldehyde (Ach) enhances accumulation of HIV-related components in hepatocytes due to their impaired degradation by lysosome and proteasome. Combined with Ach, these HIV components induce oxidative stress, abortive HIV infection of hepatocytes, and apoptotic cell death. Engulfment of hepatocyte apoptotic bodies (ABHep) by liver macrophages (Mph )and HSCs promotes pro-inflammatory and pro-fibrotic change development.

## 2. Materials and Methods

### 2.1. Reagents and Media

High glucose Dulbecco’s Modified Eagle Medium (DMEM) and fetal bovine serum were purchased from Invitrogen (Carlsbad, CA, USA), Trizol was sourced from Life Technologies, primer probes and reverse transcription polymerase chain reaction (RT-PCR) reagents were from Applied Biosystems by Thermo Fisher Scientific, (Ashveville, NC, USA). Anti-CYP2E1 was from EMD Millipore (Temecula, CA, USA). Other reagents, all analytical-grade quality, were from Sigma (St. Louis, MO, USA).

### 2.2. Cells and Treatments

We used human primary hepatocytes obtained from the Liver Tissue Cell Distribution System (Minneapolis, MN; Pittsburgh, PA; NIH Contract #HSN276201200017C). HIV was purified as described in [21]. Cells were either infected with HIV-1ADA (multiplicity of infection, MOI 0.1) for three days or with HIV combined with 50 mM ethanol treatment. We established the optimal regimen for EtOH treatment in HIV-infected hepatocytes to induce the highest apoptosis levels. In this regard, primary hepatocytes were either pre-treated with EtOH for 24 h before HIV-infection (pre-treatment), treated for the last 48 h of infection (post-treatment), or pre- and post-treatment regimens were combined. Since hepatocytes plated on collagen undergo fast de-differentiation and lose Cytochrome P4502E1 (*CYP2E1)* and Alcohol Dehydrogenase (*ADH)* expression in 24 h [22], and because the sustained expression of these ethanol-metabolizing enzymes is necessary for successful ethanol treatment, cells were plated on custom soft gels (polyelectrolyte multilayer (PEM) film coating on top of the polydimethyl siloxane surface, two-dimensional (2D) culture) to support long-term cell functionality (described in [23]).

Due to limited availability of human hepatocytes, for their experimental prototype we also used Huh7.5-CYP (RLW) cells. These cells have reduced innate immunity and can be infected with HIV. They were stably transfected to metabolize ethanol by CYP2E1, but do not express ADH. To overcome this limitation, we treated RLW cells with an acetaldehyde-generating system (AGS), which contains yeast ADH as a source of enzyme, nicotinamide adenine dinucleotide (NAD) as a co-factor, and 50 mM ethanol (EtOH) (substrate for ADH), and continuously produces physiologically relevant amounts of acetaldehyde (Ach) without toxic effects. We have characterized and successfully used these cells and AGS for HCV-based ethanol in vitro studies [24,25]. The downstream effects of AGS were validated by experiments on ethanol-treated primary hepatocytes.

Pancaspase inhibitor (PCI) from Ubiquitin-Proteasome Biotechnologies (UBPBio) Inc. (Cat#F7110, Aurora, CO, USA) was used at 10 µM for the duration of HIV + EtOH treatment. Proteasome inhibitors MG132 (Cat#F1100; 5 µM overnight) and carfilzomib (Cat#F1300; 100 nM overnight) from UBPBio, Inc. (Aurora, CO, USA), and lysosome inhibitors bafilomycin (Sigma; #B1793; 50 nM overnight) and chloroquine (Sigma; #C6698; 5, 20, 50 µM overnight) were used in this study. The HIV replication inhibitor azidothymidine (AZT) was used at a 100 mM concentration during HIV + EtOH treatment. 

### 2.3. Human Monocyte-Derived Macrophages

Monocytes were obtained from healthy donor blood elutriation. Monocyte suspensions were documented as 98% pure by criteria of cell morphology in Wright-stained cytosmears. Monocytes were cultured in 48-well plates (2 × 10^5^ cells/well) in DMEM (Sigma) with 10% heat-inactivated pooled human serum, 1% glutamine, 50 g/mL gentamicin, and/or 10 g/mL ciprofloxacin (Sigma) and human CSF-1. Culture medium was changed every three days. All tissue culture reagents were screened and found negative for endotoxin (10 pg/mL; Associates of Cape Cod, Woods Hole, MA, USA) and mycoplasma contamination (Gen-Probe II; Gen-Probe, San Diego, CA, USA). After seven days in culture, monocyte-derived macrophages (MDMs) were used for experiments.

### 2.4. Hepatic Stellate Cells (HSCs)

As the source of human hepatic stellate cells (HSCs), we used commercially available human cell line LX2 (EMD Millipore, cat SCC064) grown based on instructions from the manufacturer. 

### 2.5. Apoptotic Body (AB) Generation and Treatment Macrophages and Hepatic Stellate Cells with Apoptotic Hepatocytes

To mimic apoptosis triggered by EtOH metabolism in HIV-infected hepatocytes, HIV-infected and non-infected cells were exposed to UV light (0–100 mJ/cm^2^, 140 s) to make ABHep. In 24 h, ABs were collected from supernatant by pelleting the cells at 1500 rpm for 5 min and re-suspended in DMEM. They were exposed to MDMs and LX2-cells at a 3:1 ratio as previously described [26].

### 2.6. RNA Isolation, Real-Time Polymerase Chain Reaction, and Western Blotting

Human immunodeficiency virus-RNA (HIV RNA), interferon-stimulated genes (ISGs) with anti-viral activities such as *ISG15*, 2′-5′-oligoadenylate synthetase 1 (*OAS1*), inflammasome markers NLR family pyrin domain containing 3 (*NLRP3)*, *caspase-1,* Interleukin *(IL)-1β*, and *IL-18*, and pro-fibrotic markers collagen 1 A1 (*Col1A1*), transforming growth factor beta (*TGFβ*), and prostaglandin D receptor 2 (*PTGDR2*) were quantified by real-time PCR as previously described [27]. Total RNA was isolated from cells using Trizol Reagent. A two-step procedure was used, in which 200 ng RNA were reverse-transcribed to cDNA using the high capacity reverse transcription kit. In the second step, the cDNA was amplified using TaqMan Universal Master Mix-II with fluorescent-labeled primers (TaqMan gene expression systems). These were incubated in a Model 7500 qRT-PCR thermal cycler. The relative quantity of each RNA transcript was calculated by its threshold cycle (Ct) after subtracting that of the reference cDNA (GAPDH) as described before [27]. Reverse transcriptase (RT) activity was detected in cell supernatants as described [28]. For Western blotting (WB), cell lysates were prepared in 0.5 M ethylenediaminetetraacetic acid (EDTA), 2 M Tris, 20 mM sodium orthovanadate (Na_3_VO_4_), 200 mM tetrasodium pyrophosphate (Na_4_P_2_O_7_), 100 mM phenylmethylsulfonyl fluoride (PMSF), 1 M NaF, 20% Triton X-100, and aprotinin, pH = 7. Samples were subjected to denaturing sodium dodecyl sulfate polyacrylamide gel electrophoresis (SDS-PAGE) in polyacrylamide gels. Western blot was performed as described previously [27], blots were developed using Odyssey^®^ infrared imaging system (Li-Cor Bioscience, Lincoln, NE, USA), and the protein band was quantified using Li-Cor software. The Β-actin was used as the loading control to normalize the proteins.

### 2.7. Total HIV-1 DNA Quantification Using Semi-Nested Polymerase Chain Reaction

Total genomic DNA (gDNA) was isolated from RLW cells using the DNeasy Blood & Tissue Kit (Qiagen, Germantown, MD, USA) according to the manufacturer’s protocol. Semi-nested real-time PCR was performed as described earlier and included two rounds of PCR amplification of the HIVgag region [29]. The ACH2 cells DNA was used to construct a dilution series ranging from 105 to 10 DNA copies per sample.

### 2.8. Integrated HIV-1 DNA Quantification Using Digital-Droplet PCR (ddPCR)

For the evaluation of integrated HIV DNA, isolated gDNA was digested with restriction endonuclease enzyme and amplified with a first-round PCR amplification (95 °C for 2 min; 20 cycles of 95 °C for 15 s, 50 °C for 15 s, and 72 °C for 150 s) using 100 nM *alu* (GCCTCCCAAAGTGCTGGGATTACA) and 600 nM *gag* reverse primers GTTCCTGC TATGTCACTTCC), as described previously [29]. Further, the product of first PCR was quantified for integrated DNA by the ddPCR method. Briefly, the final PCR reaction was comprised of ddPCR supermix (Bio-Rad), 900 nM primers (sense 5′-TCAGCCCAGAAGTAATACCCATGT-3′ and antisense 5′-CACTGTGTTTAGCATGGTGTTT-3′), a 250 nM probe (FAM-ATTATCAGAAGGAGCCACCCCACAAGA3-ZEN/Iowa Black FQ), and 4 uL of first PCR product in a final volume of 20 μL, and loaded into an eight-channel disposable droplet generator cartridge (Bio-Rad). Generated droplets were then transferred into a 96-well PCR plate, heat-sealed with foil, and then amplified to endpoint using a BioRad C1000 Touch PCR cycler at 95 °C for 10 min, then 40 cycles of 94 °C for 15 s, and 60 °C for 1 min (2 °C/s ramp rate) with a final step at 98 °C for 10 min and a 4 °C hold. Plates containing amplified droplets were loaded into a QX200 droplet reader (Bio-Rad), as described previously [30]. Discrimination between negative droplets (no integrated copies) and positives (with HIV-integrated DNA) was used to estimate concentration of targets (HIV integrated copies) using QuantaSoft analysis software (Bio-Rad). The resulted copies were normalized to input RNA and represented as integrated DNA copies per μg of DNA.

### 2.9. Activities of Proteasome and Cathepsins

Proteasome chymotrypsin-like and trypsin-like activities were determined as previously reported [31] using cell lysates as the source of the enzyme in 0.1 M Tris–HCl (pH 7.5) and a final concentration of 13.3 μM N-succinyl-leu-leu-val-tyr-7-amido-4-methlycoumarin (suc-LLVY-AMC; UBPBio, Inc.) for measurement of chymotrypsin-like activity, and boc-leu-ser-thr-arg-7-amido-4-methlycoumarin (boc-LSTR-AMC; UBPBio Inc) to detect trypsin-like activity in a 200 μL reaction mixture using 96-well black bottom plates. Samples were incubated for a total of 60 min at 37 °C and during that period, read every 15 min on a spectramax plate reader at 355 nm (excitation) and 460 nm (emission), top read. Standard curve was generated based on known quantities of AMC (Sigma). Specific activity was expressed as nmols AMC/mg protein. 

Cathepsin B and L activities were assayed in cell lysates using specific fluorogenic peptide substrates, Z-arg-arg-7-amido-4-methylcoumarin hydrochloride (cathepsin B) and L-phe-arg-7-amido-4-methylcoumarin hydrochloride for cathepsin L, as described [32]. All samples were incubated at 37 °C for 20 min and fluorescence was measured every 5 min using a spectramax plate reader. Fluorescence of 7-amino-4-methylcoumarin (AMC) was measured at 370 nm (excitation) and 460 nm (emission), top read. Known quantities of AMC (Sigma) were used as standards. 

### 2.10. Validation of In Vitro Results by In Vivo Studies

For confirmation purposes, we used TK-NOG mice initially provided by Dr. Mamoru Ito, CIE, Japan and then bred at Animal Facility, UNMC, Omaha, NE. The protocol of experiments was approved by the UNMC Animal Studies Committee. Transgenic expression of herpes simplex virus thymidine kinase under albumin (Alb) promoter allows for conditional depletion of mouse hepatocytes after ganciclovir administration, which supports the expanding of transplanted human hepatocytes. We used only transgenic males that can be successfully transplanted with human hepatocytes. After aproximately four months post-transplantation, liver humanization was detected by the presence of human Alb quantified by enzyme-linked immunosorbent assay ELISA (Alb concentration 0.5–2.0 mg/mL). Mice (three mice per group) were pair-fed control and EtOH (5% *v*/*v*) Lieber De Carli diets for 10 days, then gavaged with phosphate buffer solution (PBS)/maltose dextran or EtOH on day 11 and sacrificed 9 h after gavaging, as described in detail for an chronic-acute NIH EtOH model [33]. Every other day during this feeding period they were intraperitoneally (IP)-injected with HIV-1ADA or left uninfected. 

### 2.11. Statistical Analyses

Data from at least three duplicate independent experiments are expressed as mean values ± standard error. Comparisons among multiple groups were determined by one-way ANOVA, using a Tukey post-hoc test. For comparisons between two groups, we used Student’s *t*-test. A probability value of 0.05 or less was considered significant.

## 3. Results

### 3.1. Ethanol Promotes Apoptosis in HIV-Infected Hepatocytes 

**Figure 1:**
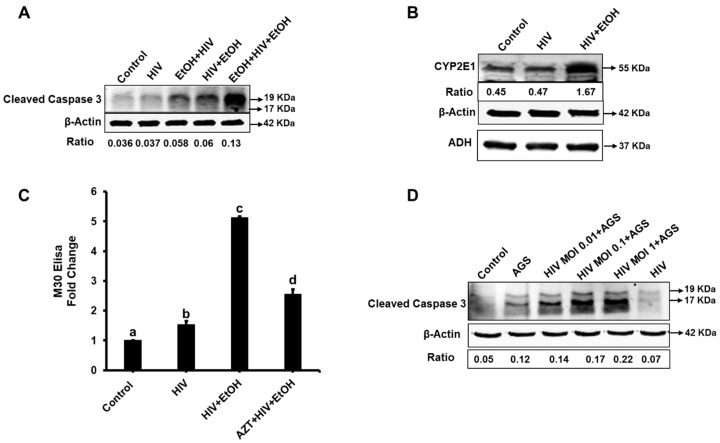
**Human immunodefeciency virus (HIV)** and ethanol induce apoptosis in hepatocytes: Data are from three independent experiments presented as means ±standard error, (SE). Bars marked with the same letter are not significantly different from each other; bars with different letters are significantly different (*p* ≤ 0.05). (**A**) Effects of various EtOH treatment regimens on caspase 3 cleavage in HIV-exposed hepatocytes (Western blotting, WB). (**B**) Prolonged expression of alcohol-metabolizing enzymes, *CYP2E1* and *ADH*, in hepatocytes plated on polyelectrolyte multilayer (PEM) gels. We cut a unnecessary group in third lane, so we joined the last lane. (**C**) M30 ELISA (apoptosis) in HIV-infected hepatocytes in the presence or absence of azidothymidine (AZT) (100 µM). (**D**) Effects of various HIV MOI on caspase 3 cleavage in RLW cells treated with an acetaldehyde-generating system (AGS).

We established the optimal regimen for EtOH treatment in HIV-infected hepatocytes to induce the highest apoptosis levels. In this regard, primary hepatocytes were either pre-treated with EtOH for 24 h before HIV-infection (pre-treatment) or treated for the last 48 h of infection (post-treatment), or pre- and post-treatment regimens were combined. Figure 1A shows that the highest caspase 3 cleavage was obtained with the combined treatment.

Plating hepatocytes on PEM soft gel allowed prolonged (up to six days) expression of ethanol-metabolizing enzymes during the time of ethanol exposure, and the combined EtOH treatment induced the stabilization of CYP2E1, but not ADH proteins in these cells (detected by WB, Figure 1B). Thus, for further experiments, we used the combined treatment of HIV-infected cells with EtOH (designated as HIV + EtOH). These caspase-3 cleavage results were confirmed by M30 ELISA demonstrating 4-fold potentiation of apoptosis in HIV-infected cells by EtOH, which was attenuated by azidothymidine (AZT) co-treatment (Figure 1C). A similar pattern was observed in an experimental prototype of hepatocytes, CYP2E1-expressing Huh7.5 (RLW) cells infected with various MOI of HIV and exposed to AGS (Figure 1D). The increase in caspase-3 cleavage by AGS was HIV-dose-dependent. 

### 3.2. HIVgag Expression in Hepatocytes Exposed to Ethanol/AGS

**Figure 2:**
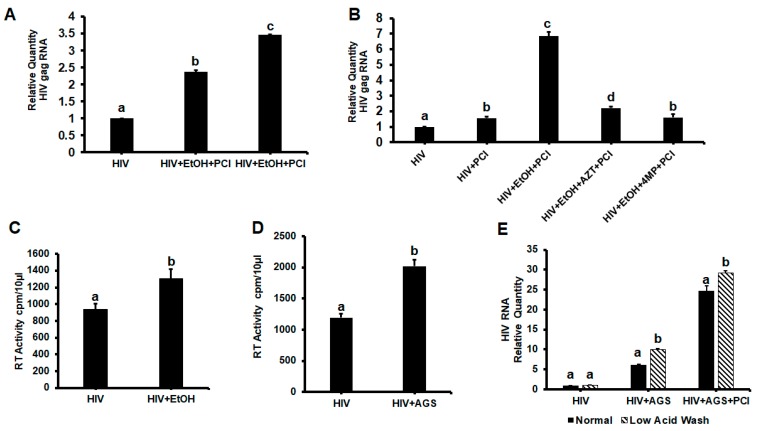
Effects of ethanol metabolism on human immunodeficiency virus gag ribonuclear acid (HIVgag RNA) expression in HIV-infected hepatocytes. All data are from three independent experiments presented as means ± SE. Bars marked with the same letter are not significantly different from each other; bars with different letters are significantly different (*p* ≤ 0.05). (**A**) Effects of pancaspase inhibitor (PCI) on HIV gag RNA in primary human hepatocytes. (**B**) Effects of (AZT and 4-methyl pyrazole (4MP) on HIV RNA in primary human hepatocytes. (**C**) Reverse transcriptase (RT) activity in supernatants of primary human hepatocytes. (**D**) RT activity in supernatants of RLW cells exposed to AGS. (**E**) The HIVgag RNA after removal of membrane-expressed structures by low acid wash in RLW cells.

Human immunodefeciency virus (HIV)-dose dependence of caspase-3 cleavage and the suppression of HIV-EtOH-induced apoptosis by AZT suggests the link between HIV infection of hepatocytes and their ability to undergo apoptosis. Thus, we measured the effects of EtOH on HIVgag RNA expression (RT-PCR) and found a 2.5-fold increase by exposure to EtOH. Since in previous experiments we observed apoptosis under these treatment conditions, we screened for HIVgag RNA levels in hepatocytes exposed to pan-caspase inhibitor (PCI) (Figure 2A). The protection of cells from apoptosis by PCI significantly upregulated the expression of HIVgag RNA, indicating that infected cells undergo apoptosis. This increase was attributed to ethanol metabolism since ethanol metabolism inhibitor, 4-methyl pyrazole (4MP) reversed the effects of ethanol on HIVgag RNA expression (Figure 2B). Ethanol only slightly enhanced low levels of reverse transcriptase (RT) activity in hepatocyte supernatants (Figure 2C) as well as in RLW cells exposed to HIV and AGS (Figure 2D). 

To exclude the possibility that HIV RNA comes from the viral particles attached to cell membrane, we treated HIV-infected RLW cells with AGS and then removed the membrane-associated binders by low acid stripping [34]. There was no reduction in HIVgag RNA by acid wash in cells protected from apoptosis with PCI (Figure 2E), indicating that HIVgag RNA detected in EtOH/AGS-treated cells is inside of cells, and thus, ethanol metabolism (mainly Ach) enhances intracellular expression of HIVgag RNA. 

### 3.3. Effect of AGS on HIV DNA Levels in RLW Cells

**Figure 3:**
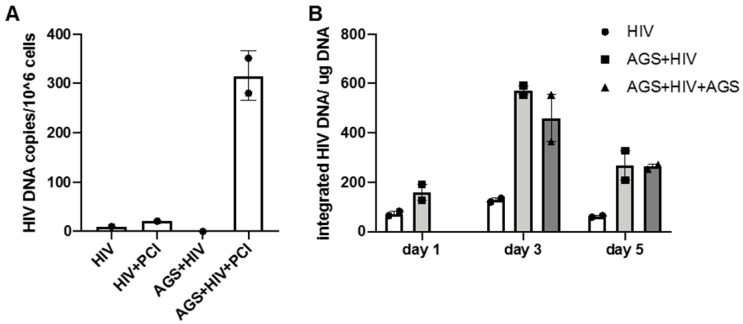
Effect of acetaldehyde generating system (AGS) on HIV-DNA levels in RLW cells. The results from duplicated representative experiment are shown in panels (**A**,**B**). (**A**) Effects of PCI on intracellular HIV DNA. (**B**) AGS increased the level of integrated HIV DNA (in the presence of PCI).

The HIV DNA was measured in the presence or absence of PCI. For these experiments, RLW cells were infected with HIV in the presence or absence of AGS and PCI. HIV DNA with AGS (pre- or combined treatment ) was detected in RLW cells in the presence of PCI. The number of detectable copies in RLW cells was measured by q-PCR. We found that AGS significantly (up to 300-fold) increased HIV DNA levels only when cells were expesosed to PCI (Figure 3A). Furthermore, AGS (both pre-treatment and combined treatment) increased the integration of HIV DNA into the cell’s genome, with the highest affects on day 3 post-infection, while at day 5 post-infectiction, less integrated HIV DNA was observed (Figure 3B).

### 3.4. Kinetics of HIV Markers in RLW Cells in Response to AGS Treatment

**Figure 4:**
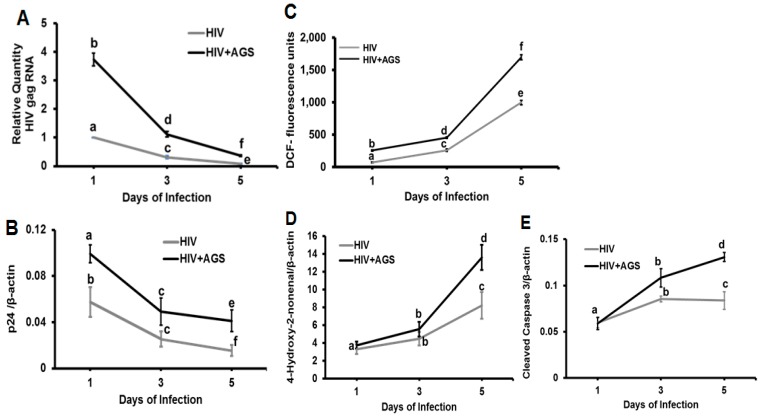
Kinetics of HIV components, oxidative stress markers and cleaved caspase-3 in HIV-infected RLW cells exposed to AGS. Data are from three independent experiments presented as means ± SE. Bars marked with the same letter are not significantly different from each other; bars with different letters are significantly different (*p* ≤ 0.05). (**A**) HIVgag RNA; (**B**) p24; (**C**) Cleaved caspase-3; (**D**) reactive oxygen species (ROS); (**E**) 4-hydroxynonenal ( 4HNE) protein adducts

To elucidate the kinetics of HIV infection in RLW cells and its connection to apoptosis, we measured the levels of HIVgag RNA and p24, adduction of cell proteins with 4-hydroxynonenal (4-HNE), and reactive oxygen species (ROS) release (2′, 7′-dichlorofluorescein fluorescence method, DCF) as well as cleaved caspase-3 at days 1, 3, and 5 post-infection (Figure 4). 

The experiments were done after one-day pretreatment of HIV-infected RLW cells with AGS in the presence of PCI (except for the set of cells for cleaved caspase-3 detection). While all tested parameters were at significantly higher levels in AGS-treated HIV-infected cells versus HIV-infected cells, the highest levels of HIV RNA and p24 were detected at day 1 post-infection, with further decrease by day 5 (*p* < 0.05) (Figure 4A,B) The kinetics of oxidative stress markers, ROS and 4-HNE-adducted protein expression, as well as cleaved caspase-3 were the opposite: we observed their increase from day 1 to day 5 of HIV-infection (*p* < 0.05). (Figure 4C–E). The levels of pro-caspase-3 were not changed. 

### 3.5. Possible Mechanisms of Up-Regulation of HIV Expression by Ethanol Metabolites

#### 3.5.1. Receptors for Viral Entry

**Figure 5:**
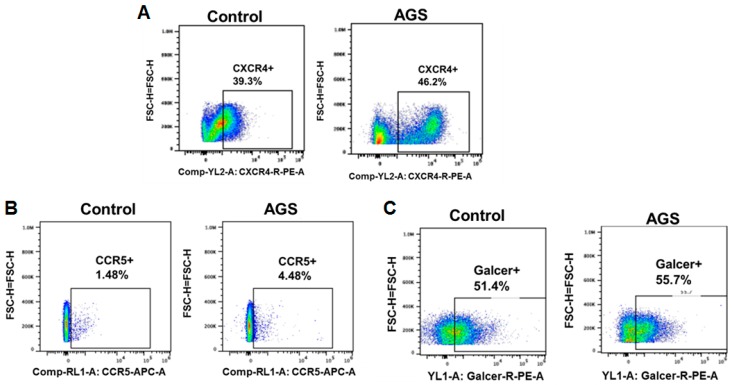
Effects of AGS on possible receptors for viral entry on hepatocytes (the results of representative experiments). (**A**) C-X-C-chemokine receptor type 4 CXCR4; (**B**) C-C-chemokine receptor type 5 (CCR5); (**C**) Galactosyl ceramide (GalCer).

In the next set of experiments, we tested a sub-hypothesis that ethanol metabolism up-regulates HIV RNA and p24 in hepatocytes due to AGS-enhanced expression of receptors for HIV entry. Since hepatocytes/hepatoma cells are CD4-negative cells, by flow cytometry we measured the effects of AGS on expression the candidate receptors for HIV entry on hepatocytes, namely, CXCR4, CCR5 and GalCer (galactosyl ceramide). The RLW cells were treated with AGS for 48 h and then expression of CXCR4, CCR5 and GalCer was detected by flow cytometry using directly labeled antibodies. As it appeared, AGS exposure to RLW cells provided minimal effects on expression of these receptors (Figure 5A–C). The highest up-regulative effect of AGS (both on % of positive cells and brightness) was observed with CXCR4 receptor reported before as a receptor for HIV entry on hepatocytes [35].

#### 3.5.2. HIV-Accumulation in Hepatocytes Due to AGS-Suppressed Degradation

**Figure 6:**
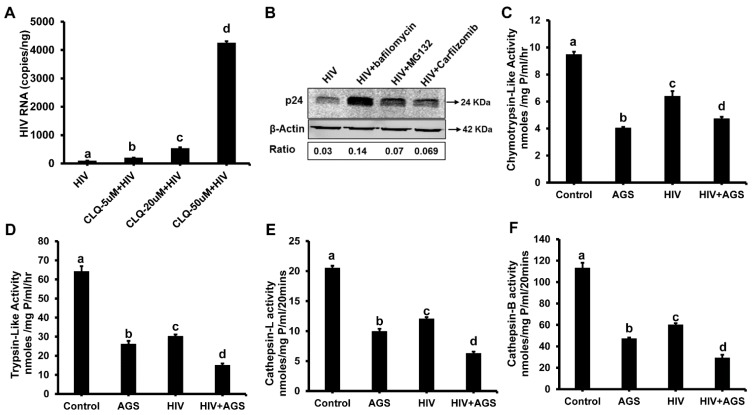
The AGS exposure promotes accumulation of HIV components by suppressing lysosome and proteasome activities in RLW cells: Data are from three independent experiments presented as means ± SE. Bars marked with the same letter are not significantly different from each other; bars with different letters are significantly different (*p* ≤ 0.05). (**A**) Chloroquine enhances expression of HIV gag RNA; (**B**) Lysosome and proteasome inhibitors stabilize p24 expression; (**C**) Chymotrypsin-like proteasome activity; (**D**) Trypsin-like proteasome activity; (**E**) Cathepsin L-activity; (**F**) Cathepsin B activity.

As another option, we cannot exclude that after HIV enters hepatocytes, its components undergo degradation by major intracellular degradation enzymes, lysosomes and proteasomes, whose activities may be suppressed by ethanol metabolism and by exposure to AGS [31,36]. To mimic this situation, we treated HIV-infected cells with the lysosome inhibitor, chloroquine, which dose-dependently increased HIV RNA levels in RLW cells (qPCR, Figure 6A). Then we studied whether the higher levels of HIV p24 expression in AGS-exposed cells (vs. HIV-infected RLW cells non-treated with AGS) detected at day 3 can be mimicked by cell exposure to the lysosome inhibitor bafilomycin or to the proteasome inhibitors MG132 and carfilzomib applied to cells one day before cell harvesting. We indeed observed stabilization of p24 by cell exposure to either lysosomal (a 4.6-fold increase) or proteasomal inhibitors (a 2.3-fold increase) (Figure 6B). Furthermore, HIV combined with AGS suppressed chymotrypsin-like and trypsin-like proteasome activities as well as activities of cathepsin L and B in RLW cells (Figure 6C–F).

### 3.6. Ethanol Metabolism-Induced Gene Activation in HIV-Infected Hepatocytes

**Figure 7:**
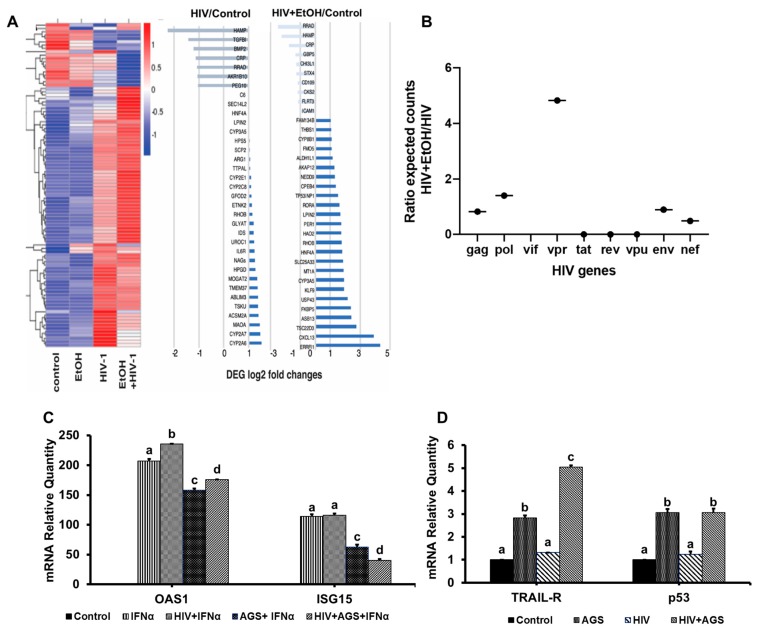
Gene activation in hepatocytes exposed to HIV and EtOH. Data are from three independent experiments presented as means ± SE. Bars marked with the same letter are not significantly different from each other; bars with different letters are significantly different (*p* ≤ 0.05): (**A**) Next-generation sequencing (NGS, heatmap); (**B**) Expression of HIV genes (NGS); (**C**) Activation of interferon-stimulated genes (ISGs) in RLW cells. (**D**) Activation of pro-apoptotic genes in RLW cells.

Since the levels of HIV RNA, p24 and HIV DNA were increased by exposure of cells to ethanol/AGS, we studied whether these treatments affected qualitative characteristics of hepatocytes, namely, gene activation profile tested by Next Generation Sequencing (NGS, DEG log2 fold change HIV vs. control and HIV + EtOH vs. control). As shown on Figure 7A, EtOH upregulates the expression of multiple genes that encode stress response (*THBS1, MT1A, CXCL13, TSC22D3, USP43, FKBP5, CPEB4, ERRFI1*) in hepatocytes.

In RNA-seq experiment (NGS), we estimated HIV gene expression levels by the abundance of mapped to HIV genome transcripts from unmapped to human genome. HIV reads were not found in uninfected control and EtOH samples. Reads of *Vif* were found only in HIV + EtOH-treated hepatocytes. Also, under ethanol treatment of hepatocytes, we detected increased HIV genes such as *Vpr* expression (Figure 7B) belonging to interferon-stimulated genes (ISG). In this regard, we additionally tested the effects of HIV and AGS exposure to RLW cells on IFNα-induced anti-viral ISGs, *OAS1*, and *ISG15*, as well as on the pro-apoptotic genes *TRAIL-R2* and *p53*. ISGs were quantified by RT-PCR in non-infected and HIV infected RLW cells treated in the presence or absence of AGS and IFNα (200 IU for the last 18 h). The combination of HIV and AGS suppressed *OAS1* and *ISG15* genes (Figure 7C). AGS and its combination with HIV increased the expression of *TRAIL-R2* and *p53* compared with uninfected and HIV-infected cells due to the up-regulative effects of AGS (Figure 7D). 

### 3.7. Hepatocyte-Derived Apoptotic Bodies (ABHep) and HIV Infection

**Figure 8:**
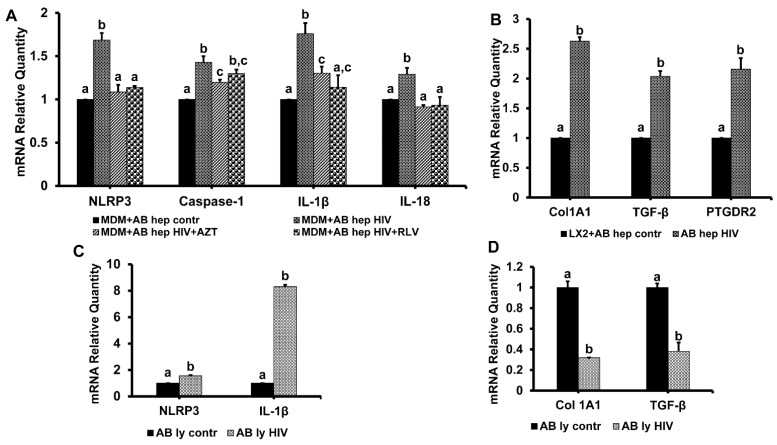
Activation of monocyte-derived macrophages (MDMs) and hepatic stellate cells (HSCs) by engulfment of ABHep. Data are from three independent experiments presented as means ± SE. Bars marked with the same letter are not significantly different from each other; bars with different letters are significantly different (*p* ≤ 0.05). (**A**) Inflammasome markers in MDMs. (**B**) Pro-fibrotic gene activation in HSCs. (**C**) Inflammasome markers in mRNA expression in lymphocytes. (**D**) Pro-fibrotic mRNAs in lymphocytes.

Although HIV- infection in hepatocytes is not productive, we observed intensive apoptosis in HIV-infected cells treated with AGS. This apoptosis leads to prominent ABHep formation. Here, we asked the question as to whether this ABHep formation from infected hepatocytes is beneficial or harmful for the liver. To mimic pro-apoptotic effects of AGS treatment, we generated ABHep from uninfected and HIV-infected RLW cells (apoptotic bodies control (ABcntr) and apoptotic bodies HIVinfected (ABHIV)) as described and characterized earlier [25]. These ABHep were incubated with MDMs for two hours and then inflammasome activation was measured based on *NLRP3*, *caspase-1*, *IL-1β*, and *IL-18* mRNA expressions. We observed the induction of all these mRNAs by engulfment of ABHIV (compared with ABcntr) (Figure 8A).

Furthermore, this effect of ABHIV was abrogated by the treatment of infected RLW cells with HIV inhibitors, AZT, or raltegravir (RLV) before ABHIV generation. 

Since HSCs can also engulf AB, which promotes their survival [37], we exposed HSC (LX2 cells) to ABcntr or ABHIV and then measured the induction of the pro-fibrotic markers collagen1 A1 (*Col1A1*), *TGFβ*, and prostaglandin D receptor 2 (*PTGDR2*) in HSCs. In all cases, there was a 2–2.5-fold increase in pro-fibrotic markers expression in response to ABHIV (compared with engulfment of ABcntr, Figure 8B). To elucidate whether pro-fibrotic activation of HSCs by ABHIV is hepatocyte-specific, in addition to ABHep, we generated ABs from HIV-infected and uninfected (control) human lymphocytes and exposed them to HSC. Unlike ABs of hepatocyte origin, engulfment of ABs from HIV-infected lymphocytes (ABlyHIV) induced pro-inflammatory activation (Figure 8C) but suppressed *Col1A1* and *TGFβ* gene activation (Figure 8D) in LX2 cells.

### 3.8. In Vivo Effects of HIV and Ethanol on Human Hepatocytes

**Figure 9:**
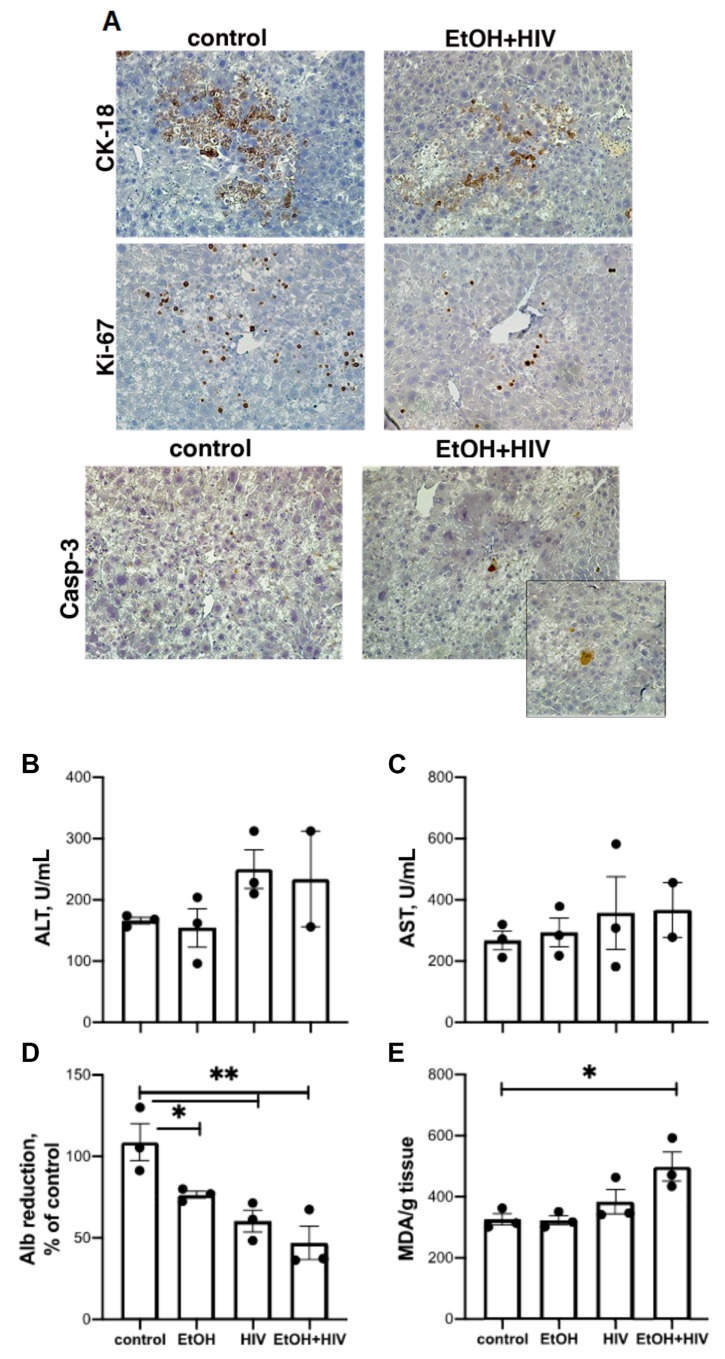
Ethanol feeding depletes human hepatocytes in HIV-exposed mice with humanized livers (pilot study). (**A**) Liver morphology (mice fed control diet vs. mice fed ethanol diet, all injected with HIV). Liver tissue was stained with the human-specific antibodies cytokeratin (CK)-18 and KI-67 as well as with anti-caspase-3. (**B**) alanine aminotransferase ALT. (**C**) aspartate aminotransferase (AST). (**D**) Human albumin (reduction, % control). (**E**) thiobarbituric acid reactive substances (TBARS).

To confirm our in vitro findings by in vivo experiments, we used TK-NOG mice. The TK-NOG males were transplanted with human hepatocytes at 10 weeks of age. Three months later the levels of human albumin (Alb) stabilized at 1.1–2.5 mg/mL. Then animals were fed control and ethanol diets as described [33]. During 10 days of pair-feeding, mice were injected with 3 × 10^5^ TCID_50_ HIV-1 i.p. every two days. Last injection was done 24 h before euthanasia. As shown in Figure 9A–D, there was human albumin reduction in the HIV + EtOH group of mice. This depletion was accompanied by the activation of oxidative stress (thiobarbituric acid reactive substances, TBARS, activity kit, Cayman Chemicals, Ann Arbor, MI, USA). While the number of human hepatocytes was significantly decreased based on human-specific Ki-67 and CK-18 staining, there were still residual caspase-3-positive (apoptotic) hepatocytes in this group not fully cleared by liver non-parenchymal cells during the feeding period.

## 4. Discussion

The liver plays a major role in the clearance of circulating viral particles in the blood, as shown for the simian immunodeficiency virus [38]. As demonstrated earlier, while hepatocytes appear to be non-permissive when affected by HIV monoinfection, their exposure to the HIV combined with second hits (HCV co-infection) may lead to increased cell death [23,39]. Here, we investigated whether HIV infects hepatocytes and if the combined insult of HIV with ethanol metabolism causes apoptotic cell death in hepatocytes. We believe that the studies on the role of ethanol metabolism in HIV-induced hepatocyte death are crucial for understanding of detrimental end-stage liver disease outcomes triggered by second hit-induced apoptosis in HIV-infected hepatocytes. 

As evident from the experiments on both primary human hepatocytes exposed to ethanol and RLW (Huh7.5-CYP) cells treated with Ach generated by AGS, HIV-dependent induction of apoptosis was attenuated by AZT (HIV reverse transcriptase inhibitor) co-treatment. In fact, apoptosis was enhanced with increasing MOI of HIV in the cells exposed to AGS, indicating the causative link between the expression of HIV in hepatocytes and apoptotic cell death. In fact, the ability of HIV antigens, namely gp120, to promote apoptosis in hepatocytes has been reported previously [40]. This apoptosis was caspase-independent [41] and was triggered via CCR5 and CXCR4 receptors on hepatocyte surface by gp120 [41,42], which does not require intracellular expression of HIV. However, in our case, when hepatocytes were infected with HIVADA, pan-caspase inhibitor suppressed the depletion of HIV RNA levels in AGS-treated cells, indicating its caspase dependence. In addition, the removal of surface cell structures by low acid wash did not reverse an increase in HIV RNA by AGS, suggesting that the observed upregulation of HIV RNA was attributable to the intracellular presence of HIV in hepatocytes but not to HIV attachment to cell membrane. 

How does acetaldehyde increase HIV levels in hepatocytes and thereby contribute to apoptosis? We found that besides HIV RNA and p24 expression, integrated HIV DNA was increased by AGS treatment. Since we were able to measure integrated HIV DNA only by very sensitive method of ddPCR, we would assume that AGS-induced HIV DNA integration into host genome is extremely low in hepatocytes. In addition, AGS treatment up-regulated integrated HIV DNA only in the presence of PCI, indicating that AGS- and HIV-induced caspase-dependent apoptosis clears up infected hepatocytes with increased levels of HIV DNA and those with HIV DNA integration. This rapid clearance of HIV-infected hepatocytes partially explains low reverse transcriptase (RT) activity in cell supernatants. In the literature, there are several very controversial reports on HIV DNA integration even in the absence of AGS: while some authors demonstrated HIV DNA integration and low levels of productive infection in hepatocytes [7,35], others did not support this evidence [43]. In fact, our experimental conditions, which allow the detection of HIV DNA integration in hepatocytes in the presence of AGS, are artificial because this phenomenon can be observed only when apoptosis in infected hepatocytes is prevented by PCI (in vitro conditions). In vivo, we faced the depletion of HIV-exposed human hepatocytes as a result of ethanol feeding, with a residual apoptosis after the feeding completion. 

To characterize the changes induced in HIV-infected hepatocytes by ethanol exposure, we performed NGS studies on HIV-infected vs. HIV-infected-ethanol-treated hepatocytes. Here, in addition to up-regulation of multiple metabolic and apoptosis-associated changes in HIV-infected hepatocytes exposed to ethanol, we observed Vpr RNA expression and an accessory viral protein known to block innate immunity factor (apolipoprotein B mRNA editing enzyme, catalytic polypeptide-like 3G gene *APOBEC3G*) to potentially increase HIV infectivity [44]. In addition, we found that other antiviral ISGs, *OAS1* and *ISG15*, were suppressed by AGS, which was associated with activation of pro-apoptotic genes, *TRAIL-R2* and *p53.* However, regardless decreased innate immunity in AGS-exposed hepatocytes, we observed no infectivity in the medium collected from HIV-infected liver cells also exposed to AGS, indicating abortive HIV replication, which in turn, may lead to apoptosis induction in these cells. Since HIV DNA integration is a prerequisite for HIV replication, this suggests that Ach increases it in a small number of hepatocytes, which is visible only if these cells do not undergo rapid apoptosis. Obviously, while exposure to Ach increases both HIV RNA and integrated HIV DNA levels, it does not make HIV-infection productive, corresponding to the previous findings observed in Ach-non-treated HIV-infected hepatocytes [45]. 

Our data provide evidence of apoptotic hepatocyte death due to accumulation of HIV components combined with the effects of ethanol metabolism, primarily, Ach. The incomplete HIV replication accompanied by accumulation of HIV DNA without integration into host genome has been shown to promote apoptosis [46]. Thus, we cannot exclude that in our study, accumulation of HIV DNA due to abortive HIV replication in hepatocytes may serve as an additional trigger of apoptosis in the case of cell co-exposure to HIV and AGS.

It is unlikely that accumulation of HIV in ethanol-metabolism-exposed hepatocytes is due to an AGS-induced increase in receptors for HIV entry. Although we observed up-regulative effect of AGS on CXCR4 receptor expression on RLW cells, it has been shown that HIV_ADA_ binds only CCR5 receptor, not CXCR4 [47,48], and thus it is very questionable whether AGS-enhanced expression of CXCR4 would increase the entry of HIV_ADA_ (which we used here) into RLW cells.

The kinetics of HIV RNA and p24 expression in liver cells demonstrate the decrease in their levels from day 1 to day 3 and further to day 5 post HIV-infection in AGS-exposed and non-exposed cells. This decrease is not due to depletion of infected cells by intensive apoptosis, which in these experiments, was prevented by co-treatment with PCI. The presence of the late HIV protein p24 at day 1–2 post-infection could be due to internalization of the viral particles in hepatocytes and prolonged p24 expression until it is degraded. In fact, lysosome was reported to participate in p24 degradation [43]. Because ethanol metabolism suppresses lysosome function and biogenesis [49], we observed higher p24 levels in hepatocytes treated with AGS. According to our findings, both lysosome and proteasome may contribute to p24 degradation since lysosomal and proteasome inhibitors applied at day 2 post infection partially slowed down the degradation of p24 by day 3, mimicking the stabilizing effects of Ach on p24 levels. The decline in cathepsin and proteasome activities by ethanol metabolism indeed has been reported before and was linked to oxidative stress induction [32,50]. In our hands, a combination of HIV-AGS provided in vitro down-regulative effects on cathepsin and proteasome activities. Importantly, according to the results of kinetic experiments, there is no direct link between HIV-specific p24 or RNA levels and increased caspase-3 cleavage, indicating that HIV components activated oxidative stress (based on increased 4HNE protein adduct expression and ROS production), which in turn, induced apoptosis in hepatocytes.

Induction of hepatocyte apoptosis upon exposure to HIV and ethanol was also confirmed by our in vivo studies on humanized mice. Multiple injection of HIV in the process of ethanol feeding of mice transplanted with human hepatocytes led to very significant depletion of human hepatocytes judged by human albumin levels. Previously, we also observed the depletion in human albumin in dual-reconstituted humanized mice transplanted with human hepatocytes and mismatched human hematopoietic/progenitor cells infected with HIV [29]. However, upon ethanol feeding, significant HIV-induced cell death (human albumin depletion) occurred even in the absence of human immune system. The increased expression of the oxidative stress marker TBARS accompanied by the presence of residual apoptotic cells and HIV DNA in hepatocytes as the only transplanted HIV-infectable human cells was observed in these ethanol-fed mice.

**Figure 10:**
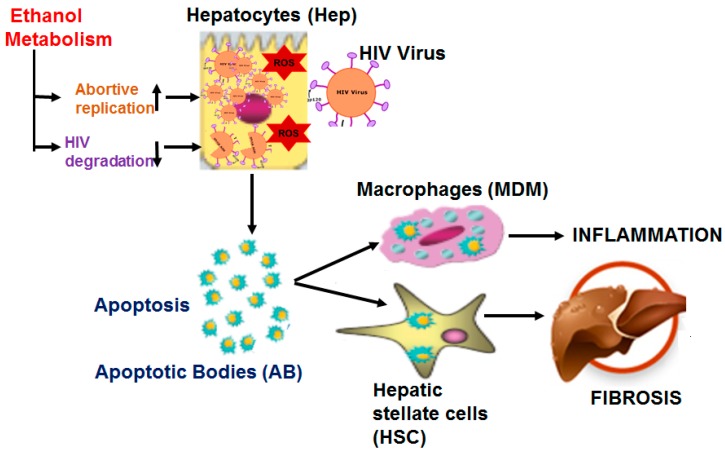
Ethanol metabolism promotes apoptosis in HIV-infected hepatocytes, causing liver inflammation and fibrosis development. Ethanol metabolism suppresses lysosome and proteasome activities in HIV-infected hepatocytes and causes accumulation of HIV components, finally leading to apoptosis induction and abortive HIV replication. Apoptotic hepatocytes that contain viral components are engulfed by macrophages (MDMs) and HSCs, thereby promoting activation of inflammasome in MDMs and activation of pro-fibrotic genes in HSCs, leading to liver inflammation and fibrosis development.

What is the consequence of intensive hepatocyte apoptosis driven by HIV and alcohol metabolism? Apoptosis serves as a method of HIV-infected hepatocyte elimination, but is it a truly beneficial event? Exposure of MDM to ABs from HIV-infected hepatocytes caused activation of inflammasome, while in HSC, it induced the activation of pro-fibrotic genes. Thus, the engulfment of ABs generated from HIV-infected hepatocytes by non-parenchymal cells contributes to pathogenesis of HIV-infection in the liver, suggesting that ethanol metabolism-induced apoptosis in HIV-infected hepatocytes may promote liver inflammation and fibrosis development. These properties of ABs are pathogen and hepatocyte-specific; however, the nature of these events is not quite clear yet. Our major in vitro findings are summarized in Figure 10.

## 5. Conclusions

We conclude that ethanol metabolism by slowing down the degradation of HIV components by lysosomes and proteasomes causes accumulation of HIV patterns in hepatocytes to induce oxidative stress and apoptotic cell death. Clearance of these HIV-infected apoptotic bodies by liver non-parenchymal cells promotes inflammasome activation in macrophages and pro-fibrotic gene activation in hepatic stellate cells, thereby contributing to liver disease progression.

## References

[1] Debes J.D., Bohjanen P.R., Boonstra A. (2016). Mechanisms of accelerated liver fibrosis progression during hiv infection. J. Clin. Transl. Hepatol..

[2] Pascual-Pareja J.F., Caminoa A., Larrauri C., Gonzalez-Garcia J., Montes M.L., Diez J., Grande M., Arribas J.R. (2009). Haart is associated with lower hepatic necroinflammatory activity in hiv-hepatitis c virus-coinfected patients with cd4 cell count of more than 350 cells/microl at the time of liver biopsy. AIDS.

[3] Mata-Marin J.A., Gaytan-Martinez J., Grados-Chavarria B.H., Fuentes-Allen J.L., Arroyo-Anduiza C.I., Alfaro-Mejia A. (2009). Correlation between hiv viral load and aminotransferases as liver damage markers in hiv infected naive patients: A concordance cross-sectional study. Virol. J..

[4] Penton P.K., Blackard J.T. (2014). Analysis of hiv quasispecies suggests compartmentalization in the liver. AIDS Res. Hum. Retrovir..

[5] Sterling R.K., Chiu S., Snider K., Nixon D. (2008). The prevalence and risk factors for abnormal liver enzymes in hiv-positive patients without hepatitis B or C coinfections. Dig. Dis. Sci..

[6] Kooij K.W., Wit F.W., van Zoest R.A., Schouten J., Kootstra N.A., van Vugt M., Prins M., Reiss P., van der Valk M., Group A.G.C.S. (2016). Liver fibrosis in hiv-infected individuals on long-term antiretroviral therapy: Associated with immune activation, immunodeficiency and prior use of didanosine. AIDS.

[7] Kong L., Cardona Maya W., Moreno-Fernandez M.E., Ma G., Shata M.T., Sherman K.E., Chougnet C., Blackard J.T. (2012). Low-level hiv infection of hepatocytes. Virol. J..

[8] Pandrea I., Happel K.I., Amedee A.M., Bagby G.J., Nelson S. (2010). Alcohol’s role in hiv transmission and disease progression. Alcohol. Res. Health.

[9] Koziel M.J., Peters M.G. (2007). Viral hepatitis in hiv infection. N. Engl. J. Med..

[10] Barve S., Kapoor R., Moghe A., Ramirez J.A., Eaton J.W., Gobejishvili L., Joshi-Barve S., McClain C.J. (2010). Focus on the liver: Alcohol use, highly active antiretroviral therapy, and liver disease in hiv-infected patients. Alcohol. Res. Health.

[11] Chaudhry A.A., Sulkowski M.S., Chander G., Moore R.D. (2009). Hazardous drinking is associated with an elevated aspartate aminotransferase to platelet ratio index in an urban hiv-infected clinical cohort. HIV Med..

[12] Wada N.I., Jacobson L.P., Margolick J.B., Breen E.C., Macatangay B., Penugonda S., Martinez-Maza O., Bream J.H. (2015). The effect of haart-induced hiv suppression on circulating markers of inflammation and immune activation. AIDS.

[13] Schnabl B., Brenner D.A. (2014). Interactions between the intestinal microbiome and liver diseases. Gastroenterology.

[14] Bansal M.B., Blackard J.T., Sherman K.E. (2012). Effects of hiv on liver cell populations. Hiv and Liver Disease.

[15] Cao Y.Z., Dieterich D., Thomas P.A., Huang Y.X., Mirabile M., Ho D.D. (1992). Identification and quantitation of hiv-1 in the liver of patients with aids. AIDS.

[16] Lin W., Weinberg E.M., Tai A.W., Peng L.F., Brockman M.A., Kim K.A., Kim S.S., Borges C.B., Shao R.X., Chung R.T. (2008). Hiv increases hcv replication in a tgf-beta1-dependent manner. Gastroenterology.

[17] Nunez M. (2006). Hepatotoxicity of antiretrovirals: Incidence, mechanisms and management. J. Hepatol..

[18] Qin F., Jiang J., Qin C., Huang Y., Liang B., Xu Y., Huang J., Xu Z., Ning C., Liao Y. (2019). Liver damage in patients living with hiv on antiretroviral treatment with normal baseline liver function and without hbv/hcv infection: An 11-year retrospective cohort study in Guangxi, China. BMJ Open.

[19] Cummins N.W., Badley A.D. (2010). Mechanisms of hiv-associated lymphocyte apoptosis: 2010. Cell Death Dis..

[20] Finkel T.H., Tudor-Williams G., Banda N.K., Cotton M.F., Curiel T., Monks C., Baba T.W., Ruprecht R.M., Kupfer A. (1995). Apoptosis occurs predominantly in bystander cells and not in productively infected cells of hiv- and siv-infected lymph nodes. Nat. Med..

[21] Gorantla S., Che M., Gendelman H.E. (2005). Isolation, propagation, and hiv-1 infection of monocyte-derived macrophages and recovery of virus from brain and cerebrospinal fluid. Methods Mol. Biol..

[22] Godoy P., Hewitt N.J., Albrecht U., Andersen M.E., Ansari N., Bhattacharya S., Bode J.G., Bolleyn J., Borner C., Bottger J. (2013). Recent advances in 2d and 3d in vitro systems using primary hepatocytes, alternative hepatocyte sources and non-parenchymal liver cells and their use in investigating mechanisms of hepatotoxicity, cell signaling and adme. Arch. Toxicol..

[23] Ganesan M., Dagur R.S., Makarov E., Poluektova L.I., Kidambi S., Osna N.A. (2018). Matrix stiffness regulate apoptotic cell death in hiv-hcv co-infected hepatocytes: Importance for liver fibrosis progression. Biochem. Biophys. Res. Commun..

[24] Ganesan M., Zhang J., Bronich T., Poluektova L.I., Donohue T.M., Tuma D.J., Kharbanda K.K., Osna N.A. (2015). Acetaldehyde accelerates hcv-induced impairment of innate immunity by suppressing methylation reactions in liver cells. Am. J. Physiol. Gastrointest Liver Physiol..

[25] Ganesan M., Natarajan S.K., Zhang J., Mott J.L., Poluektova L.I., McVicker B.L., Kharbanda K.K., Tuma D.J., Osna N.A. (2016). Role of apoptotic hepatocytes in hcv dissemination: Regulation by acetaldehyde. Am. J. Physiol. Gastrointest Liver Physiol..

[26] Ganesan M., Poluektova L.Y., Enweluzo C., Kharbanda K.K., Osna N.A. (2018). Hepatitis c virus-infected apoptotic hepatocytes program macrophages and hepatic stellate cells for liver inflammation and fibrosis development: Role of ethanol as a second hit. Biomolecules.

[27] Ganesan M., Tikhanovich I., Vangimalla S.S., Dagur R.S., Wang W., Poluektova L.I., Sun Y., Mercer D.F., Tuma D., Weinman S.A. (2018). Demethylase jmjd6 as a new regulator of interferon signaling: Effects of hcv and ethanol metabolism. Cell. Mol. Gastroenterol. Hepatol..

[28] Gendelman H.E., Orenstein J.M., Martin M.A., Ferrua C., Mitra R., Phipps T., Wahl L.A., Lane H.C., Fauci A.S., Burke D.S. (1988). Efficient isolation and propagation of human immunodeficiency virus on recombinant colony-stimulating factor 1-treated monocytes. J. Exp. Med..

[29] Dagur R.S., Wang W., Cheng Y., Makarov E., Ganesan M., Suemizu H., Gebhart C.L., Gorantla S., Osna N., Poluektova L.Y. (2018). Human hepatocytes depletion in the presence of hiv-1 infection in dual reconstituted humanized mice. Biol. Open.

[30] Dagur R.S., Branch-Woods A., Mathews S., Joshi P.S., Quadros R.M., Harms D.W., Cheng Y., Miles S.M., Pirruccello S.J., Gurumurthy C.B. (2019). Human-like nsg mouse glycoproteins sialylation pattern changes the phenotype of human lymphocytes and sensitivity to hiv-1 infection. BMC Immunol..

[31] Ganesan M., Krutik V.M., Makarov E., Mathews S., Kharbanda K.K., Poluektova L.Y., Casey C.A., Osna N.A. (2019). Acetaldehyde suppresses the display of hbv-mhc class i complexes on hbv-expressing hepatocytes. Am. J. Physiol. Gastrointest Liver Physiol..

[32] Thomes P.G., Ehlers R.A., Trambly C.S., Clemens D.L., Fox H.S., Tuma D.J., Donohue T.M. (2013). Multilevel regulation of autophagosome content by ethanol oxidation in hepg2 cells. Autophagy.

[33] Bertola A., Mathews S., Ki S.H., Wang H., Gao B. (2013). Mouse model of chronic and binge ethanol feeding (the niaaa model). Nat. Protoc..

[34] Kameyama S., Horie M., Kikuchi T., Omura T., Tadokoro A., Takeuchi T., Nakase I., Sugiura Y., Futaki S. (2007). Acid wash in determining cellular uptake of fab/cell-permeating peptide conjugates. Biopolymers.

[35] Xiao P., Usami O., Suzuki Y., Ling H., Shimizu N., Hoshino H., Zhuang M., Ashino Y., Gu H., Hattori T. (2008). Characterization of a cd4-independent clinical hiv-1 that can efficiently infect human hepatocytes through chemokine (c-x-c motif) receptor 4. AIDS.

[36] Donohue T.M., Osna N.A. (2003). Intracellular proteolytic systems in alcohol-induced tissue injury. Alcohol. Res. Health.

[37] Jiang J.X., Mikami K., Venugopal S., Li Y., Torok N.J. (2009). Apoptotic body engulfment by hepatic stellate cells promotes their survival by the jak/stat and akt/nf-kappab-dependent pathways. J. Hepatol..

[38] Zhang L., Dailey P.J., Gettie A., Blanchard J., Ho D.D. (2002). The liver is a major organ for clearing simian immunodeficiency virus in rhesus monkeys. J. Virol..

[39] Hu S., Ghabril M., Amet T., Hu N., Byrd D., Yang K., Vuppalanchi R., Saxena R., Desai M., Lan J. (2014). Hiv-1 coinfection profoundly alters intrahepatic chemokine but not inflammatory cytokine profiles in hcv-infected subjects. PLoS ONE.

[40] Babu C.K., Suwansrinon K., Bren G.D., Badley A.D., Rizza S.A. (2009). Hiv induces trail sensitivity in hepatocytes. PLoS ONE.

[41] Vlahakis S.R., Villasis-Keever A., Gomez T.S., Bren G.D., Paya C.V. (2003). Human immunodeficiency virus-induced apoptosis of human hepatocytes via cxcr4. J. Infect. Dis..

[42] Balasubramanian A., Ganju R.K., Groopman J.E. (2006). Signal transducer and activator of transcription factor 1 mediates apoptosis induced by hepatitis c virus and hiv envelope proteins in hepatocytes. J. Infect. Dis.

[43] Fromentin R., Tardif M.R., Tremblay M.J. (2011). Inefficient fusion due to a lack of attachment receptor/co-receptor restricts productive human immunodeficiency virus type 1 infection in human hepatoma huh7.5 cells. J. Gen. Virol.

[44] Wang X., Ao Z., Chen L., Kobinger G., Peng J., Yao X. (2012). The cellular antiviral protein apobec3g interacts with hiv-1 reverse transcriptase and inhibits its function during viral replication. J. Virol..

[45] Park I.W., Fan Y., Luo X., Ryou M.G., Liu J., Green L., He J.J. (2014). Hiv-1 nef is transferred from expressing t cells to hepatocytic cells through conduits and enhances hcv replication. PLoS ONE.

[46] Martinez-Picado J., Zurakowski R., Buzon M.J., Stevenson M. (2018). Episomal hiv-1 DNA and its relationship to other markers of hiv-1 persistence. Retrovirology.

[47] Kolchinsky P., Mirzabekov T., Farzan M., Kiprilov E., Cayabyab M., Mooney L.J., Choe H., Sodroski J. (1999). Adaptation of a ccr5-using, primary human immunodeficiency virus type 1 isolate for cd4-independent replication. J. Virol..

[48] Westervelt P., Gendelman H.E., Ratner L. (1991). Identification of a determinant within the human immunodeficiency virus 1 surface envelope glycoprotein critical for productive infection of primary monocytes. Proc. Natl. Acad. Sci. USA.

[49] Chao X., Wang S., Zhao K., Li Y., Williams J.A., Li T., Chavan H., Krishnamurthy P., He X.C., Li L. (2018). Impaired tfeb-mediated lysosome biogenesis and autophagy promote chronic ethanol-induced liver injury and steatosis in mice. Gastroenterology.

[50] Osna N.A., Donohue T.M. (2013). Cyp2e1-catalyzed alcohol metabolism: Role of oxidant generation in interferon signaling, antigen presentation and autophagy. Cytochrome P450 2E1: Its Role in Disease and Drug Metabolism.

